# Enhancement of the expression of HCV core gene does not enhance core-specific immune response in DNA immunization: advantages of the heterologous DNA prime, protein boost immunization regimen

**DOI:** 10.1186/1479-0556-7-7

**Published:** 2009-06-08

**Authors:** Ekaterina Alekseeva, Irina Sominskaya, Dace Skrastina, Irina Egorova, Elizaveta Starodubova, Eriks Kushners, Marija Mihailova, Natalia Petrakova, Ruta Bruvere, Tatyana Kozlovskaya, Maria Isaguliants, Paul Pumpens

**Affiliations:** 1 Latvian Biomedical Research and Study Centre, Ratsupites 1, Riga, LV-1067, Latvia; 2 Swedish Institute of Infectious Disease Control, SE-17182 Stockholm, Sweden; 3 Pasteur Institute, 197101 St Petersburg, Russia; 4 Microbiology and Tumorbiology Center, Karolinska Institutet, 17177 Stockholm, Sweden; 5 D.I. Ivanovsky Institute of Virology, 123098 Moscow, Russia

## Abstract

**Background:**

Hepatitis C core protein is an attractive target for HCV vaccine aimed to exterminate HCV infected cells. However, although highly immunogenic in natural infection, core appears to have low immunogenicity in experimental settings. We aimed to design an HCV vaccine prototype based on core, and devise immunization regimens that would lead to potent anti-core immune responses which circumvent the immunogenicity limitations earlier observed.

**Methods:**

Plasmids encoding core with no translation initiation signal (pCMVcore); with Kozak sequence (pCMVcoreKozak); and with HCV IRES (pCMVcoreIRES) were designed and expressed in a variety of eukaryotic cells. Polyproteins corresponding to HCV 1b amino acids (aa) 1–98 and 1–173 were expressed in *E. coli*. C57BL/6 mice were immunized with four 25-μg doses of pCMVcoreKozak, or pCMV (I). BALB/c mice were immunized with 100 μg of either pCMVcore, or pCMVcoreKozak, or pCMVcoreIRES, or empty pCMV (II). Lastly, BALB/c mice were immunized with 20 μg of core aa 1–98 in prime and boost, or with 100 μg of pCMVcoreKozak in prime and 20 μg of core aa 1–98 in boost (III). Antibody response, [^3^H]-T-incorporation, and cytokine secretion by core/core peptide-stimulated splenocytes were assessed after each immunization.

**Results:**

Plasmids differed in core-expression capacity: mouse fibroblasts transfected with pCMVcore, pCMVcoreIRES and pCMVcoreKozak expressed 0.22 ± 0.18, 0.83 ± 0.5, and 13 ± 5 ng core per cell, respectively. Single immunization with highly expressing pCMVcoreKozak induced specific IFN-γ and IL-2, and weak antibody response. Single immunization with plasmids directing low levels of core expression induced similar levels of cytokines, strong T-cell proliferation (pCMVcoreIRES), and antibodies in titer 10^3^(pCMVcore). Boosting with pCMVcoreKozak induced low antibody response, core-specific T-cell proliferation and IFN-γ secretion that subsided after the 3rd plasmid injection. The latter also led to a decrease in specific IL-2 secretion. The best was the heterologous pCMVcoreKozak prime/protein boost regimen that generated mixed Th1/Th2-cellular response with core-specific antibodies in titer ≥ 3 × 10^3^.

**Conclusion:**

Thus, administration of highly expressed HCV core gene, as one large dose or repeated injections of smaller doses, may suppress core-specific immune response. Instead, the latter is induced by a heterologous DNA prime/protein boost regimen that circumvents the negative effects of intracellular core expression.

## Background

Globally, an estimated 170 million people are chronically infected with hepatitis C virus (HCV), and 3 to 4 million persons are newly infected each year [1,2]. The human immune system has difficulties in clearing the virus in either the acute, or chronic phase of the infection with up to 40% of patients progressing to cirrhosis and liver failure [3-6]. Extensive studies have unraveled important reliable correlates of viral clearance [7-11]. This, together with the growing need to diminish the magnitude of HCV associated liver disease served as a basis for intensive HCV vaccine research. A series of HCV vaccine candidates have moved into clinical trials [11]. One such is the peptide vaccine IC41 consisting of a panel of MHC class I and class II restricted epitopes adjuvanted by poly-L-arginine administered to healthy volunteers [12] and to chronic HCV patients including non-responders to the standard therapy [13,14]. Another therapeutic vaccine employed peptides chosen individually for their ability to induce the strongest *in vitro *cellular response [15]. In a further vaccine trial, chronic hepatitis C patients received the recombinant HCV envelope protein E1 [16]. The first clinical trial of an HCV DNA vaccine consisting of a codon-optimized NS3/4A gene administered to chronic hepatitis C patients is currently ongoing (CHRONVAC-C^®^; ; ).

So far, none of the peptide or protein vaccines were able to induce a significant improvement in the health conditions of chronic HCV patients, or a significant decrease of HCV RNA load, specifically if compared to the conventional IFN-based therapy [13,15,16]. The vaccine trials have, however, demonstrated that when achieved, HCV RNA decline in the vaccine recipients correlates with induction of strong IFN-gamma T-cell response [13]. Such a response can best be recruited by DNA vaccines, either alone or with the aid of heterologous boosts [11,17]. Indeed, vaccination of chimpanzees showed the ability to elicit effective immunity against heterologous HCV strains using T-cell oriented HCV genetic vaccines that stimulated only the cellular arm of the immune system [17,18].

An attractive target for HCV vaccine is the nucleocapsid (core) protein [19-21]. It is highly conserved among various HCV genotypes with amino acid homology exceeding 95% [21,22]. Core binds and packages the viral genomic RNA, regulates its translation [23-26] and drives the production of infectious viruses [27-29]. Core contributes to HCV persistence also indirectly by interfering with host cell transcription, apoptosis, lipid metabolism, and the development of immune response [30-33]. Extermination of core expressing cells and inhibition of the activity of extracellular core (non-enveloped particles containing HCV RNA [34]) could be highly beneficial.

Ideally, HCV core could be eliminated by a specific vaccine-induced immune response. It is a strong immunogen with anti-core immune response evolving very early in infection [35,36]. Early and broad peripheral and intrahepatic CD8+ T-cell and antibody response to core/core epitopes is registered in chimpanzees controlling HCV infection HCV, but not in chimpanzees that become chronically infected [37-39]. In mice, potent experimentally induced anti-core immune response conferred partial protection against challenge with core expressing recombinant vaccinia virus [40]. However, despite high immunogenicity in the natural infection, core does not perform well as an immunogen, specifically if introduced as naked DNA [2,41-43]. Attempts to enhance core immunogenicity by targeting HCV core protein to specific cellular compartments [44], co-immunization with cytokine expressing plasmids [2,41], adjuvants as CpG [45], or truncated core gene versions [46] had limited or no success.

Prime-boost strategies have been used to increase immune responses to a number of DNA vaccines. Immunization regimens comprised of a DNA prime and a viral vector boost for instance for vaccinia virus [47,48], adenovirus [49], fowlpox [50,51], and retrovirus [52]. Priming with DNA and boosting with protein is another promising approach. This regimen has been studied for HIV [53,54], hepatitis C virus [55,56], anthrax [57], *Mycobacteria *[58,59], *Streptococcus pneumoniae *[60] and BVDV [61]. DNA vaccines and recombinant protein vaccines utilize different mechanisms to elicit antigen-specific responses. Due to the production of antigen in transfected cells of the host, a DNA vaccine induces robust T-cell responses, which are critical for the development of T-cell-dependent antibody responses [62]. DNA immunization is also highly effective in priming antigen-specific memory B cells. In contrast, a recombinant protein vaccine is generally more effective at eliciting antibody responses than cell-mediated immune responses and may directly stimulate antigen-specific memory B cells to differentiate into antibody-secreting cells, resulting in production of high titer antigen-specific antibodies [63]. Therefore, a DNA prime plus protein boost is a complementary approach that overcomes each of their respective shortcomings. Certain improvement of the immune response was reached after co-delivery of HCV core DNA and recombinant core [2,40,64]. In this study, we have shown that in DNA immunization, poor core-specific immune response can be a consequence of high levels of intracellular core expression, and that such a response can be improved by using low-expressing core genes, or single core gene primes in combination with recombinant core protein boosts.

## Methods

### Plasmids for expression of HCV core

Region encoding aa 1–191 of HCV core was reverse-transcribed and amplified from HCV 1b isolate 274933RU (GeneBank accession #AF176573) [65] using oligonucleotide primers: sense GATCCAAGCTTATGAGCACGAATCC and antisense GATCCCTCGAGTCAAGCGGAAGCTGG containing recognition sites of HindIII and XhoI restriction endonucleases. The amplified DNA was cleaved with HindIII/XhoI and inserted into pcDNA3 (Invitrogen, USA) cleaved with HindIII/XhoI resulting in pCMVcore. Region encoding aa 1–191 of HCV core was also reverse-transcribed and amplified from HCV isolate 274933RU using another set of primers that carried Kozak consensus sequence sense AGCTGCTAGCGCCGCCACCATGAGCACGAATCCT and antisense GATCGTTAACTAAGCGGAAGCTGGATGG primers containing recognition sites of restriction endonucleases NheI and XhoI, respectively. The amplified DNA was cleaved with NheI/KspAI and inserted into the plasmid pCMVE2/p7-2 [66] cleaved with NheI/XhoI, resulting in pCMVcoreKozak. The region corresponding to HCV 5'UTR, and coding sequences for aa 1–809 was reverse-transcribed and amplified from HCV 1b isolate AD78P1 (GeneBank accession #AJ132997) [67], kindly provided by Prof. M. Roggendorf (Essen, Germany) using sense-GACCCAAGCTTCGTAGACCGTGCACCAT and antisense CATGCTCGAGTTAGGCGTATGCTCG primers. The amplified DNA was cleaved with HindIII/XhoI and inserted into pcDNA3 cleaved with HindIII/XhoI resulting in pCMVcoreIRES. HCV 274933RU core differed from HCV AD78P1 core in positions 70 (H versus R), 75 (T versus A), and 147 (V versus T), respectively.

Growth of pcDNA3, pCMVcore, pCMVcoreKozak, and pCMVcoreIRES was accomplished in the *E. coli *strain DH5alpha. Plasmid DNA was extracted and purified by Endo Free plasmid Maxi kit (Qiagen GmbH, Germany). The purified plasmids were dissolved in the phosphate buffered saline (PBS) and used for *in vitro *expression assays and for DNA immunization.

### Cell transfection, lysis and Western-blotting

BHK-21, COS-7, and NIH3T3 cells were seeded into plates (3 × 10^5 ^cells/well) and transfected by plasmid DNA (2 μg) using Lipofectamine (GIBCO-BRL, Scotland) or ExGen 500 (Fermentas, Lithuania) as described by the manufacturers. HCV core expression was analyzed 24, 48 and 72 h post transfection. Cells were lysed for 10 min at 0°C in the buffer containing 50 mM Tris-HCl, pH 7.5, 1 mM EDTA, 1 mM PMSF and 1% NP-40. Lysates were cleared by 10 min centrifugation at 6000 g, resolved by 12% SDS-PAAG, and transferred to PVDF membranes (Amersham Pharmacia Biotech, Ireland). HCV core expression was detected by immunostaining with polyclonal rabbit anti-core antibodies [68], and secondary horseradish peroxidase (HRP)-conjugated anti-rabbit immunoglobulins (Amersham Pharmacia Biotech, Ireland) followed by ECL™ detection (ECL Plus, Amersham Pharmacia Biotech, Ireland).

### Quantification of core expression in mouse cells

NIH3T3 cells were transfected with either pcDNA3, pCMVcore, pCMVcoreKozak, pCMVcoreIRES, or pEGFP-N1 (Clontech, CA, USA). The percent of transfection was evaluated by counting the number of GFP expressing cells per 500 transfected NIH3T3 cells using a fluorescence Leica DM 6000 microscope (Leica Camera AG, Germany). Cells were harvested 48 h post-transfection, counted, and 10^4 ^cells were lysed in 2× SDS Sample buffer. Lysates and samples containing 1 to 50 ng of recombinant core aa 1–173 (corresponding to p21) were run simultaneously on 12% SDS-PAAG and transferred onto PVDF membrane for calibration. Blots were blocked overnight in PBS-T with 5% non-fat dry milk, stained with polyclonal anti-core antibodies #35-6 (1:5000) followed by the secondary anti-rabbit HRP-conjugated antibodies (DAKOPatts AB, Denmark). Signals were detected using the ECL™ system (Amersham Pharmacia Biotech, Ireland). X-ray films were scanned, and processed using Image J software . The data are presented as the Mean Grey Values (MGV). The core content was quantified by plotting the MGV of each sample onto a calibration curve prepared using recombinant core aa 1–173. After core detection, blots were striped according to the ECL protocol and re-stained with monoclonal anti-tubulin antibodies (Sigma, USA) and secondary anti-mouse HRP-conjugated antibodies (DAKOPatts AB, Denmark). Core content per transfected cell was evaluated after accounting for the percent of transfection and normalization to the tubulin content per well.

### Immunofluorescence staining

BHK-21 cells were seeded on the chamber slides (Nunc International, Denmark) and transfected as above. 24 h post transfection, the slides were dried, fixed with acetic acid and ethanol (1:3) for 15 min and rinsed thoroughly in distilled water. Fixed cells were re-hydrated in PBS, and incubated for 24 h at 4°C with anti-HCV core rabbit polyclonal antibodies (1:50) in the blocking buffer (PBS with 2.5 mM EDTA and 1% BSA). Secondary antibodies were goat anti-rabbit immunoglobulins labeled with TRITC (1:200; DAKO, Denmark). Slides were then mounted with PermaFluor aqueous mounting medium (Immunon, Pa., USA) and read using a fluorescence microscope.

### Recombinant HCV-core proteins and core-derived peptides

Peptides covering core amino acids 1–18, 1–20, 23–43, 34–42, 133–142 and a control peptide TTAVPWNAS from gp41 of HIV-1 were purchased from Thermo Electron GmbH (Germany). Core proteins representing aa 1–152 of HCV 274933RU and aa 1–98, and 1–173 of AD78P1 were expressed in *E. coli *and purified by chromatography as was described earlier [69,70]. Purified proteins were dissolved in PBS.

### Mice and immunization

The following immunizations were performed:

#### Scheme I

Groups of 12 female 8-week old C57BL/6 mice (Stolbovaya, Moscow Region, Russia) were immunized with a total of 100 μg of pCMVcoreKozak, or empty vector, split into four i.m. injections done with 3–4 week intervals. Control mice were mock-immunized with PBS.

#### Scheme II

Female 6–8 week old BALB/c mice (Animal Breeding Centre of the Institute of Microbiology and Virology, Riga) had injected into their Tibialis anterior (TA), 50 μl of 0.01 mM cardiotoxin (Latoxan, France) in sterile 0.9% NaCl five days prior to immunization. Groups of 6 to 7 mice were immunized with a single 100 μg dose of either pCMVcoreIRES, or pCMVcore, or pCMVcoreKozak, or empty vector, all dissolved in 100 μl PBS, applied intramuscularly (i.m.) into the cardiotoxin-treated TA. Control mice were left untreated.

#### Scheme III

Groups of 5 to 6 female 6–8 week old BALB/c mice pretreated with cardiotoxin, were injected i.m. with 100 μg of pCMVcoreKozak and boosted three weeks later with 20 μg of core aa 1–98 in PBS, or primed and boosted subcutaneously with 20 μg of core aa 1–98 in PBS. Control animals were left untreated.

### ELISA

Mice were bled from retro-orbital sinus prior to, and 2 to 3 weeks after each immunization, or 5 weeks post a single gene immunization (in Scheme II). Peptides corresponding to core aa 1–20, 23–43 or 133–142 were coated onto 96-well MaxiSorp plates (Nunc, Denmark), and recombinant core aa 1–98, 1–152, or 1–173, on the 96-well PolySorp plates (Nunc, Denmark). Coating was done overnight at 4°C in 50 mM carbonate buffer, pH 9.6 at antigen concentration of 10 μg/ml. After blocking with PBS containing 1% BSA for 1 h at 37°C, serial dilutions of mouse sera were applied on the plates and incubated for an additional hour at 37°C. Incubation was followed by three washings with PBS containing 0.05% Tween-20. Afterwards, plates were incubated with the horseradish peroxidase-conjugated anti-mouse antibody (Sigma, USA) for 1 h at 37°C, washed, and substrate OPD (Sigma, USA) added for color development. Plates were read on an automatic reader (Multiscan, Sweden) at 492 nm. ELISA performed on plates coated with core aa 1–98, 1–152, or 1–173 showed similar results (data not shown). Immune serum was considered positive for anti-core antibodies whenever a specific OD value exceeded, by at least two-fold, the signals generated by: pre-immune serum reacting with core-derived antigen, and by immune serum reacting with BSA-coated plate, the assays performed simultaneously.

### T-cell proliferation assay

For T-cell proliferation tests, mice were sacrificed and spleens were obtained two weeks after each immunization in Scheme I; and three and five weeks after the last immunization in Schemes II and III. Murine splenocytes were harvested using red blood cell lysing buffer (Sigma, USA), single cell-suspensions were prepared in RPMI 1640 supplemented with 2 mM L-Glutamine and 10% fetal calf serum (Gibco BRL, Scotland) at 6 × 10^6 ^cells/ml. Cell were cultured in U-bottomed microculture plates at 37°C in a humidified 5% CO_2 _chamber (Gibco, Germany). Cell stimulation was performed with peptides representing core aa 1–20, 23–43, 34–42 and recombinant core aa 1–98, 1–152, and aa 1–173 at dilutions to 3.1, 6.25, 12.5, 25.0, 50.0, and 100 μg/ml, all in duplicate. Concanavalin A (ConA) was used as a positive control at 2 μg/ml. Cells were grown for 72 h, after which [^3^H]-thymidine (1 μCi per well; Amersham Pharmacia Biotech, Ireland) was added. After an additional 18 h, cells were harvested onto cellulose filters and the radioactivity was measured on a beta counter (Beckman, USA). The results were presented as stimulation indexes (SI), which were calculated as a ratio of mean cpm obtained in the presence and absence of a stimulator (protein or peptide). Empty-vector immunized and control mice showed SI values of 0.8 ± 0.4. SI values ≥ 1.9 were considered as indicators of specific T-cell stimulation.

### Quantification of cytokine secretion

For detection of cytokines, cell culture fluids from T-cell proliferation tests were collected, for IL-2 – 24 h, and for IL-4 and IFN-γ – 48 h post the on-start of T-cell stimulation. Detection of cytokines in the cell supernatants was performed using commercial ELISA kits (Pharmingen, BD Biosciences, CA, USA) according to the manufacturers' instructions.

## Results

### Cloning and expression

Plasmids were constructed encoding core of HCV 1b isolate 274933RU without translation initiation signals (pCMVcore); and with Kozak translation initiation signal (pCMVcoreKozak). Core with viral translation initiation signal IRES taken in the natural context was derived from HCV 1b isolate AD78P1 [67]. Viral cores had a minimal sequence difference in positions 70, 75, and 147, all three cases representing homologous substitutions.

**Figure 1:**
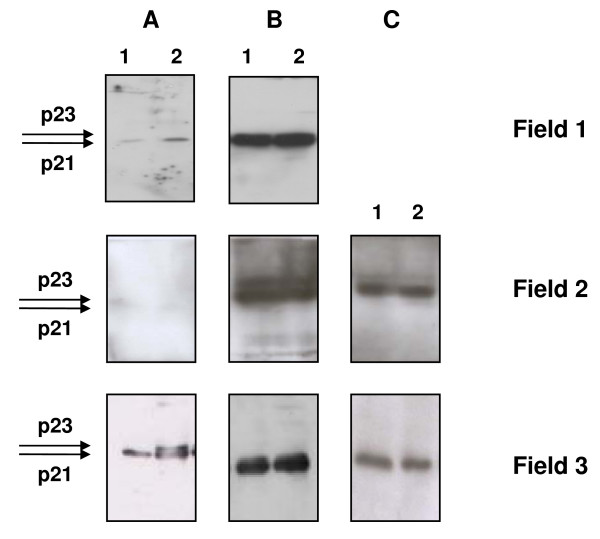
**Expression of HCV core proteins**. Expression of HCV core protein directed by pCMVcore (A), pCMVcoreKozak (B), pCMVcoreIRES (C) in COS-7 cells 72 h post-transfection (Field 1); in BHK-21 cells 48 h (Field 2) and 72 h post transfection (Field 3). Transfection with the recommended amount (lane 1), and two-fold excess of transfection reagent (lane 2).

**Figure 2:**
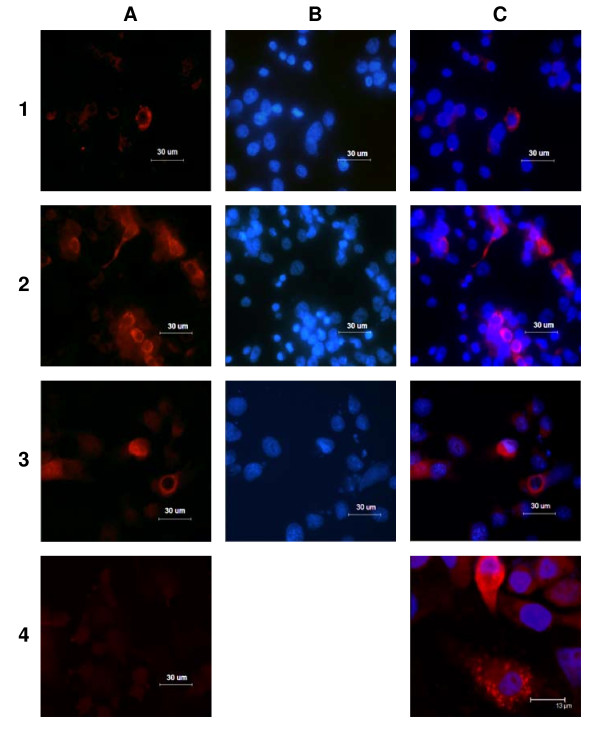
**Immunocytochemical detection of HCV core proteins**. Immunocytochemical detection of HCV core expression after transfection of BHK-21 cellswith pCMVcore (A1–C1), pCMVcoreKozak (A2–C2, C4), pCMVcoreIRES (A3–C3); nontransfected BHK-21 cells (A4). Immunostaining for HCV core protein using rabbit polyclonal anti-HCVcore antibody 35-7 as primary and TRITC-conjugated anti-rabbit immunoglobulin (IgG) as secondary antibody (panel A); nuclear staining by DAPI (panel B); overlay of A and B (panel C); negative control (nontransfected BHK-21 cells) after staining (A4). Fluorescent images A1–4, B1–3, C1–3 and A4 were taken with Leica DM 6000 B microscope and a Leica DFC 480 camera, and confocal image of cells transfected with pCMVcoreKozak and showing cytoplasmic, granular distribution (C4) with a Leica TCS SP2 SE.

Expression from these plasmids was tested both *in vitro *and in cell cultures. Plasmids pCMVcore and pCMVcoreKozak were used as the templates for the T7-driven mRNA transcription; mRNA was translated *in vitro *in the rabbit reticulocyte lysate system. Both mRNAs generated a translation product of approximately 23 kDa corresponding to the molecular mass of unprocessed HCV core (p23; data not shown). Next, core-expressing vectors were used to transfect a series of mammalian cell lines. Western blotting of BHK-21 and COS-7cells transfected with pCMVcore, pCMVcoreKozak and pCMVcoreIRES using core-specific antibodies demonstrated an accumulation of proteins with the expected molecular mass of 21 kDa that corresponds to core aa 1–171 cleaved from the full-length core by cellular proteases [71,72] (Fig. 1). Minimal amounts of p23 were also detected, specifically after transfections of BHK-21 with pCMVcore and pCMVcoreIRES (Fig. 1). The overall level of HCV core synthesis in BHK-21 cells was somewhat higher than in COS-7 cells (Fig. 1). In both cell lines, the highest level of core expression was achieved with pCMVcoreKozak (Fig. 1, 2). All cells expressing core and immunostained with core-specific antibodies demonstrated cytoplasmic granular staining characteristic of the processed p21 form of HCV core [72-74] (Fig. 2).

**Figure 3:**
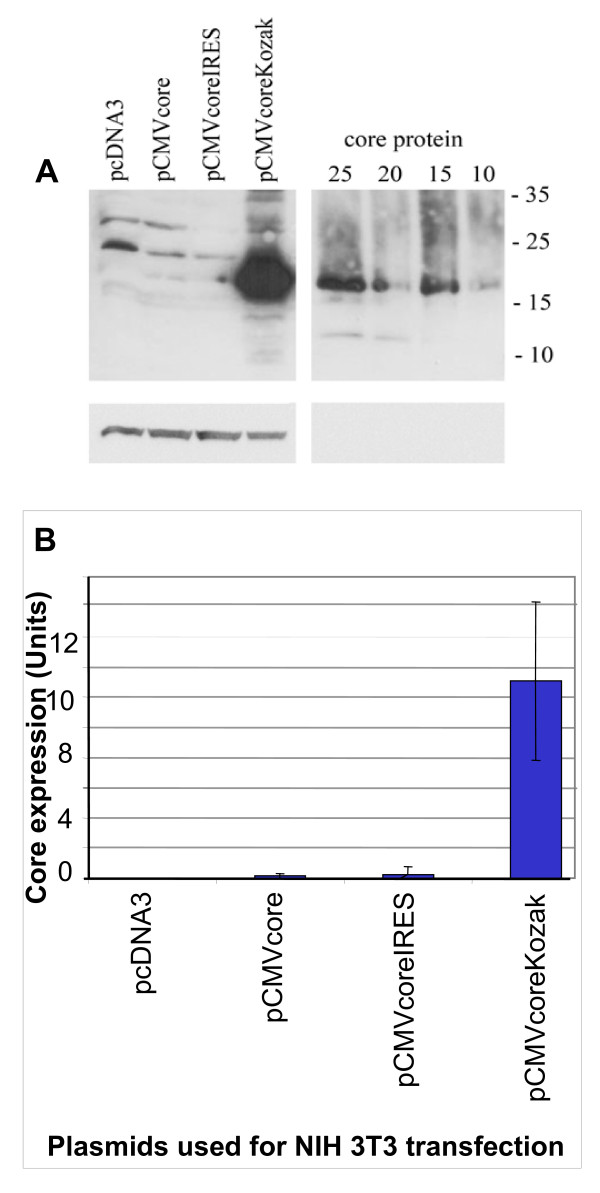
**Expression of proteins in mouse fibroblasts NIH 3T3 48 h post-transfection**. A calibration curve was prepared using recombinant core protein aa 1–173 loaded in amounts of 25, 20, 15, and 10 ng per well (lanes 5, 6, 7 and 8, respectively). Western blotting was done using rabbit anti-core antibodies [82] (A). ECL photos of blots were scanned, and images were quantified with ImageJ software . The results of quantification of HCV core expression in four independent experiments (B).

The expression capacity of the vectors was quantified in murine fibroblasts to reproduce DNA immunization that was to be done in mice. Core expression was assessed on Western blots of SDS-PAAG resolving lysates of NIH3T3 transfected with core expressing and control plasmids (Fig. 3A and 3B). Images of Western blots were processed using the ImageJ software and individual bands were represented in arbitrary units (Mean Grey Values, MGV). Their correspondence to core quantity was established using calibration curves built with the use of recombinant core aa 1–173 (see Additional file 1) after normalization to the percent of transfection and protein content of the samples. Plasmid pCMVcore with no translation initiation signals provided the lowest level of core expression (Fig. 3B). IRES promoted a two-fold increase, and the Kozak sequence, a 35-fold increase of core expression with > 15 ng of protein produced per expressing cell (Fig. 3B).

### Immunization of mice with HCV core DNA

All plasmids were purified by standard protocols in accordance with a GLP practice for preparation of DNA vaccines, and used in a series of mouse immunization experiments.

#### HCV core DNA in priming and boosts

**Figure 4:**
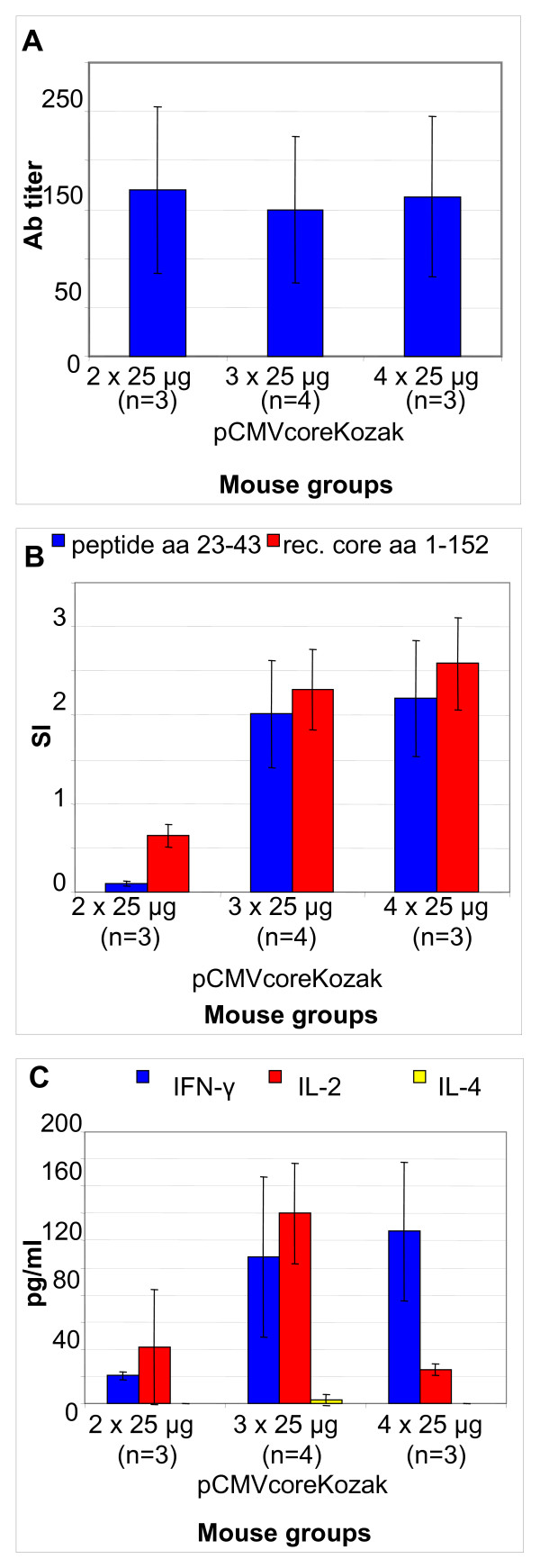
**Core-specific immune response in C57Bl/6 mice receiving 2 (2 × 25 μg), 3 (3 × 25 μg), and 4 (4 × 25 μg) injections of pCMVcoreKozak**. Maximal titers of IgG specific to recombinant core and a peptide representing core aa 1–20 (A); T-cell proliferation measured as the stimulation index (SI) in response to HCV core (1–173) and a peptide pool covering aa 23–43 of HCV core (B); cytokine secretion (pg/ml) in response to recombinant HCV core (C). Data are average values for mice assayed at a given time point.

Plasmid directing the highest level of core expression was selected and a pilot experiment defining the strategy of DNA immunization was performed. C57BL/6 mice were immunized four times with 25 μg of pCMVcoreKozak, and core-specific antibody and cellular responses were evaluated. No specific response was registered after the 1^st ^injection (data not shown). The immune response generated after the following three boosts is illustrated by Fig. 4. Three injections of 25 μg led to no increase of core-specific IgG response over the initial levels achieved after the first two plasmid injections (Fig. 4A). Three plasmid injections generated a better T-cell proliferative response to core and core-derived peptides than two. However, the response could not be boosted further (Fig. 4B). IFN-γ and IL-2 response to core was also boostable. However again, no boosting was seen after the initial two pCMVcoreKozak injections (Fig. 4C). Furthermore, the repeated injections led to a significant decrease of IL-2 secretion in response to splenocyte stimulation by recombinant core and peptides representing core N-terminus (p < 0.05; Fig. 4B, and data not shown). Core-specific IL-4 secretion was not detected.

Thus, the development of core-specific immune responses occurred within six weeks after the on-start of immunization; repeated boosts with HCV core gene did not lead to a significant enhancement of core-specific immunity.

#### HCV core DNA as a single injection

In the next series of experiments, we selected BALB/c mice as a strain that is expected to support a better Th2-type response with stronger antibody production [75]. Plasmid pCMVcore Kozak was given as a single 100 μg injection with the effect of repeated intramuscular DNA boosts substituted by pre-treatment of the injection sites by cardiotoxin [76]. T-cell proliferative response, antibody production and cytokine secretion were monitored two and five weeks after immunization.

**Figure 5:**
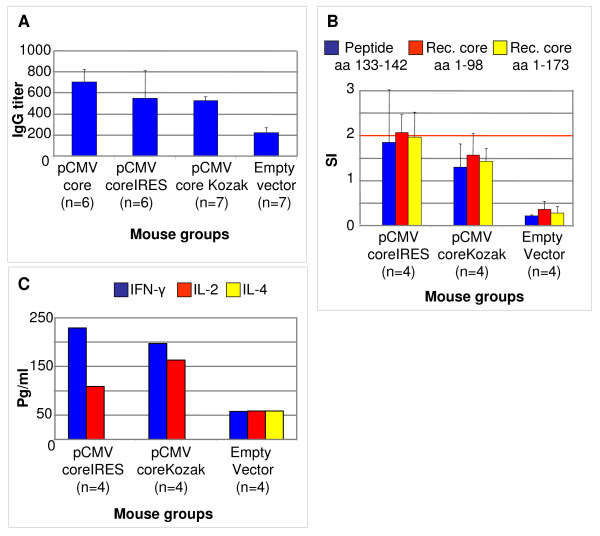
**Core-specific immune response in BALB/c mice immunized with one 100 μg dose of pCMVcoreKozak (n = 7), pCMVcoreIRES (n = 6), pCMVcore (n = 6), and empty vector (n = 7)**. The highest titers of IgG specific to core reached throughout immunization (A); T-cell proliferation measured as the stimulation index (SI) in response to recombinant HCV cores aa 1–98 and aa 1–173 and peptide representing HCV core aa 133–142 (B); the levels of core-specific IFN-γ, IL-2, and IL-4 secretion in the cell culture fluids collected after splenocyte stimulation with HCV core aa 1–98 (C). Cytokine secretion in BALB/c mice is represented by the amounts detected in the pooled cell culture fluids from the T-cell proliferation test; therefore, no standard deviations are presented.

Significant responses in the form of core-specific IFN-γ and IL-2 secretion exceeding the background levels in empty-vector-immunized mice were detected five weeks after a single administration of HCV core gene (Fig.5). Immunization generated no core-specific T-cell response and a low titer of core-specific IgG. Antibody response against HCV core has already been shown to develop slowly [46], mirroring the development of anti-core antibody response in HCV infected individuals [77]. Here as well, a slow increase in the level of anti-core antibodies was observed 35 days after a single gene injection as compared to levels detected at day 21 (data not shown). There was no difference between BALB/c and C57BL/6 mice with respect to core-specific IFN-γ secretion (Fig. 4C versus Fig. 5C), or core-specific IgG production (p > 0.05 Mann Whitney U-test; Fig. 4A versus Fig. 5A and Additional file 1).

#### High core gene expression affects core-specific immune response

The magnitude of anti-core response suggested that the increase of HCV core gene dose either by one-time large dose injection, or by repeated injections of smaller doses, did not significantly enhance core-specific immunity. To delineate if that could be influenced by core expression level, BALB/c mice were immunized with a single dose of low-expressing core genes with no translation initiation signals (pCMVcore), or with IRES (pCMVcoreIRES). The results were compared to immunization with core gene regulated by the Kozak sequence (pCMVcoreIRES) (Fig. 5). The T-cell proliferative response to core- and core-derived peptides was stronger in mice immunized with pCMVcoreIRES (Fig. 5). The highest anti-core IgG response was raised in mice immunized with pCMVcore that directed the lowest level of HCV core expression (Fig. 3; Fig. 5A). It was significantly higher than the antibody response induced by pCMVcoreKozak (p < 0.05); the immune response in pCMVcoreIRES-immunized mice was intermediate (Fig. 5A). The T-cell proliferative response to core- and core-derived peptides was stronger in mice immunized with pCMVcoreIRES (Fig. 5B; p < 0.05). While IL-2 secretion was somewhat higher in mice immunized with highly expressing pCMVcoreKozak, both DNA immunogens provided a similar level of core-specific IFN-γ secretion (Fig. 5C).

#### Heterologous DNA prime-protein boost regimen

**Figure 6:**
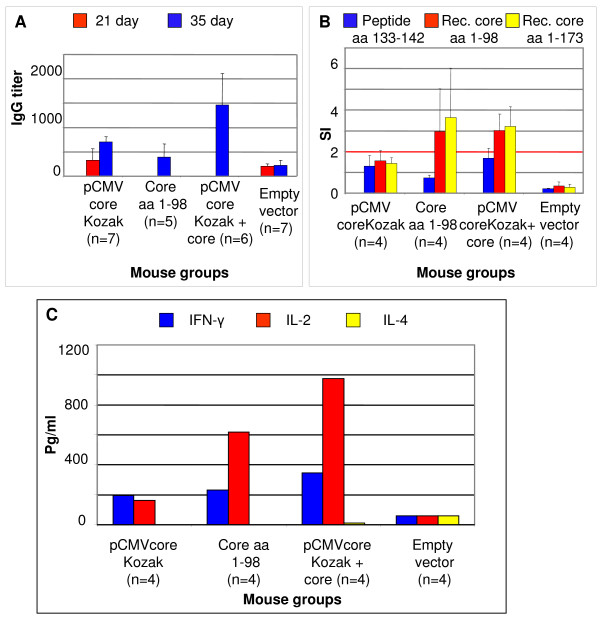
**Core-specific immune response in BALB/c mice immunized with the recombinant N-terminal domain of HCV core aa 1–98 alone (n = 5); with pCMVcoreKozak (n = 7); and primed with pCMVcoreKozak and boosted with HCV core aa 1–98 (n = 6)**. The kinetics of IgG response to core aa 1–98 (A);-T-cell proliferation measured as the stimulation index (SI) in response to HCV core aa 1–98, recombinant core aa 1–173, and a peptide covering HCV core aa 133–142 (B); levels of core-specific IFN-γ and IL-2 secretion (pg/ml) in the pooled cell culture after splenocyte stimulation with HCV core aa 1–98 or aa 1–173 (C).

We aimed to see if core-specific immune response could be enhanced without increasing core gene doses, but instead by using the heterologous prime-boost immunization regimens. HCV core protein aa 1–98 and pCMVcoreKozak were used to immunize BALB/c mice either separately, or in the DNA prime-protein boost regimen. A high titer of core-specific antibodies was achieved only after the heterologous boost (Fig. 6A). The heterologous regimen effectively induced a proliferative response, both in SI values (p levels 0.034, Mann Whitney U-test) and in the number of positive T-cell proliferation tests (p level 0.014; Fig. 6B); and potent core-specific IFN-γ and IL-2 secretion (Fig. 6C). Core-specific IL-4 secretion was, in all cases, very low.

**Figure 7:**
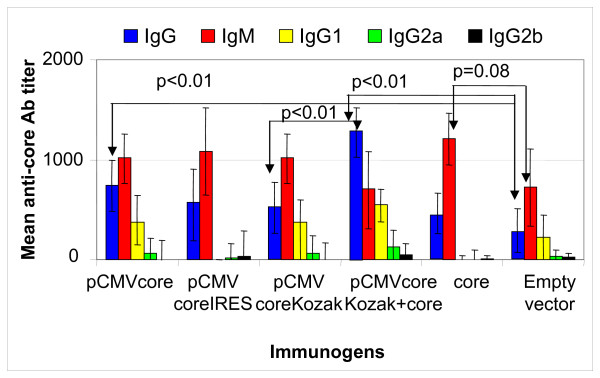
**Spectrum of core-specific immune response**. The kinetics of IgG, IgM, IgG1, IgG2a and IgG2b response to core aa 1–98 after immunization with different immunogens.

Heterologous regimen induced significant anti-core antibody production (Fig. 6A). Sera of mice primed with pCMVcoreKozak and boosted with core aa 1–98 were analysed for the presence of anti-core antibodies of IgG, IgG1, IgG2a, IgG2b and IgM subclasses, and the results were compared to seroreactvivity in mice immunized with single injection of core or core expressing plasmids (Fig. 7). Mice primed with pCMVcoreKozak and boosted with core protein had significantly higher levels of anti-core IgG than mice immunized with pCMVcoreKozak (p = 0.0006, Mann-Whitney U-test) or pCMVcore (p = 0.002) (immunization with pCMVcore gave higher level of IgG than immunization with pCMVcoreKozak, p < 0.05). Group with heterologous prime/boost regimen had also an increased levels of anti-core IgG1, although the difference with the control group did not reach the level of significance (p < 0.1). Antibodies of IgG2a or IgG2b subclasses were not found. Low specific anti-core IgM were observed only in mice immunized with recombinant core aa 1–98 (p < 0.1; Fig. 7). It was higher than in mice primed with core DNA and boosted with core protein (p level 0.05). At the same time, core-immunized mice had no anti-core IgG1 or IgG2 (Fig. 7). Thus, the heterologous core DNA prime/core protein boost regimen preferentially induced anti-core IgG, while protein immunization triggered mostly low-level anti-core IgM.

## Discussion

The immune response in DNA immunization depends on the amount of antigen produced from the immunogen *in vivo *as predetermined by the gene dose and by the gene capacity to direct an efficient antigen expression [78,79]. Normally, the response increases with the increase of the dose and efficacy of gene expression (for examples, see [80-83]). However, the DNA immunogen used here encodes not just a structural component of the virus, but also a pathogenic factor. HCV core protein interacts with a broad range of cellular proteins and influences numerous host cell functions [31,32,39,71,84,85]. Of importance for HCV vaccine design was to find to what extent the immune response to HCV core in DNA immunization is influenced (positively, or negatively) by the level of core expression as determined by gene dose (i), and gene expression efficacy (ii).

The first issue was addressed in a series of immunizations in which the same dose of HCV core was given as a single or split into multiple injections. We and others have earlier observed that repeated HCV core gene boosts do not lead to an enhancement of core-specific immune response [42,46,68]. On the contrary, both core-specific IFN-γ and IL-2 production [68] and anti-core antibody response [2,46,64] appear to be down-regulated. Here as well, the overall comparison between immunizations carried out by single and multiple core gene injections in different mouse strains demonstrated that the outcomes of immunization with one 100 μg versus two to four 25 μg core gene doses were quite similar (Fig. 4; see also the summary in Additional file 2). Furthermore, antibody response was not boosted; T-cell proliferative response and core-specific IFN-γ secretion could not be boosted beyond the levels reached after the initial two injections, and core-specific IL-2 secretion even appeared to be suppressed. Thus, core-specific immune response can be achieved after single DNA immunization, while repeated core gene administration may actually suppress core-specific immunity.

The issue of translation efficacy was assessed in single-dose immunizations with plasmids directing different levels of HCV core expression. There are different ways to increase the level of gene expression efficacy such as the use of strong promoters, optimal species-specific codons, and manipulations with RNA folding [78,78,79]. An important factor is the efficacy of translation initiation. In the CAP-dependent translation of mammalian genes it is determined by sequences flanking the AUG initiator codon. High levels of translation are achieved with the Kozak sequence, a guanine at position +4 and an adenine at -3 from AUG [86,87]. The alternative mechanisms of initiation site selection on eukaryotic cellular and viral mRNAs, also of HCV, include the translation initiation from IRESs (internal ribosome entry sites/segments) [88]. Located in the 5'-UTR region of viral genome, HCV IRES is optimized to hijack the ribosomes and translation factors from the host for the translation of HCV polyprotein [89]. Core is tightly involved in the IRES-mediated regulation of HCV translation with several regulatory signals localized in both core protein and core coding sequence [24,90,91]. Thus, the 5'-end of HCV genome incorporating 5'-UTR and core coding sequence were harmonized during evolution to provide for the levels of core expression essential for the virus.

Both CAP and IRES translation initiation options were employed in the design of core DNA immunogens. Eukaryotic expression vectors were constructed encoding core of HCV 1b without translation initiation signal (pCMVcore), core preceded by the 5'-UTR of HCV 1b isolate AD (pCMVcoreIRES), and core preceded by the consensus Kozak sequence (pCMVcoreKozak). The latter directed the expression of 35-fold more core than the gene devoid of the translation initiation signals, and 16-fold more core than the gene regulated by IRES. However, despite a considerable difference in the core expression capacity, Kozak- and IRES-regulated DNA immunogens induced similar levels of core-specific IFN-γ secretion (Fig. 5C). More so, while IL-2 secretion was somewhat higher in mice immunized with highly expressing pCMVcoreKozak, a T-cell proliferative response to core- and core-derived peptides was stronger in mice immunized with pCMVcoreIRES (Fig. 5B). Thus, high core expression levels did not promote a better core specific cellular response.

DNA-based immunization can induce potent antibody response including virus neutralizing antibodies [92-96]. However, no significant antibody response has ever been induced in core gene immunization unless it was followed by the protein boost [2,64]. Anti-core antibody titers obtained here after immunization with both CAP- and IRES-regulated core genes were also low. Interestingly, however, significantly higher titers of anti-core antibodies were obtained in mice that received the least expressed core gene devoid of any translation regulation signals (pCMV core; Figs. 5A, 7). Thus, the use of highly expressing HCV core DNA did not promote an effective core-specific antibody response.

Altogether, this points to possible adverse effects of the high-level as well as of the prolonged HCV core gene expression. We have additional data in support of this concept from immunization of C57BL/6 mice with a synthetic truncated HCV core gene devoid of HCV core nucleotide-sequence dependent regulatory signals. The latter expressed HCV 1b core at five to six-fold lower levels than the viral full-length core gene [97], but nevertheless, was capable of inducing potent core-specific cellular and antibody response [98].

DNA immunization with antigens co-expressed in natural virus infection can result in inhibition of both protein expression and specific immune response [99]. More so, pathological effects were reported of the repeated immunization with certain microbial genes, for example the hsp60 gene of *Mycobacterium *that causes necrotizing bronchointerstitial pneumonia and bronchiolitis in healthy mouse recipients, and multifocal regions of cellular necrosis in lungs when applied therapeutically [100,101]. HCV core is the factor of HCV pathogenicity. It activates cellular and viral promoters [102], induces ER- and mitochondrial stress [103,104], regulates apoptosis [105,106], tumorigenesis [107,108], and induces abnormal lipid metabolism [109]. In experimental systems, core expression leads to the development of diverse pathological effects including CD4+ T-cell depletion, liver steatosis, insulin resistance, and hepatocellular carcinoma [33,110]. One of the notable although controversial features is the capacity of HCV core to suppresses host immunity [32,39,84,85]. These features of HCV core may explain why here a better immune response was achieved after single immunization with vectors providing for comparatively low HCV core expression.

Altogether, this points to the necessity to devise alternative immunization regimens that would help to circumvent possible adverse effects of HCV core.

Many approaches can be pursued, with DNA vaccination combined with heterologous protein or recombinant viral boosts considered as the most promising [11]. The principle of this strategy is to prime T-cells to be antigen-specific and then, upon repeated exposure to a specific antigen, induce a rapid T-cell expansion. In heterologous boosts, the encoded antigen is delivered in a different form/different vehicle [111]. DNA plasmids are perfectly fit for priming since they are internalized by antigen presenting cells and can induce antigen presentation via MHC class I or class II. Such heterologous regimens can be effective when infection occurs with both viral particles and virus-infected cells, and neither cellular, nor antibody response is sufficient for sterilizing protection or viral clearance, if acting alone. This approach may help to circumvent the negative effects of intracellular core expression. Indeed, here, the heterologous DNA-prime/protein boost strategy was shown to be advantageous to both immunizations with core DNA and with the recombinant core protein (Figs. 6, 7). Protein alone performed even worse than single DNA injections (Figs. 6, 7). Only the heterologous DNA-prime-protein boost regimen induced a significant core-specific antibody production and potent T-cell response of mainly Th1-profile. This may be beneficial since most correlates of spontaneous HCV clearance are Th1-oriented [32,39,84,85].

## Conclusion

This data suggests that the administration of highly expressed HCV core gene, as well as repeated core gene injections may hamper core-specific immune response. The boosting effect of repeated core gene injections is transient as it disappears with subsequent injections. One possible way to enhance core-specific response is to deliver limited intracellular amounts of core, either by giving lower plasmid doses, or by giving vectors with low expression efficacy. An additional option is the use of heterologous DNA prime/protein boost regimen that leads to potent immune response of a mixed Th1/Th2-type. We are currently testing if transient HCV core gene expression and acquisition of anti-HCV core immunity affect the immune status and functionality of the immune system in gene recipients.

## Competing interests

The authors declare that they have no competing interests.

## Authors' contributions

IS and EK constructed plasmids and screened their immunogenicity; EA did experiments on expression and wrote a draft of the manuscript; ES and EI carried out quantifications of core expression; DS and NP did immunological experiments; RB conducted the immunocytochemistry; MI was involved with the immunological experiments, statistical evaluations and worked with the manuscript; TK and PP give useful scientific advice and revised the manuscript. All authors read and approved the final manuscript.

## Supplementary Material

Additional file 1 **Establishment of calibration curves for quantification of core expression *in vitro***. Recombinant core aa 1–173 in serial dilutions in the range of 10 to 25 ng (A), 10 to 100 ng (B), or 12.5 to 100 ng (C) was loaded on 15% SDS-PAAG and resolved by gel electrophoresis together with the study samples. Proteins were transferred to PVDF membrane and subjected to Western blotting with core-specific rabbit antibodies, and secondary anti-rabbit HRP-conjugated antibodies (DAKOPatts). Signals were registered using X-ray films and ECL detection system, images were saved, scanned, and signal of individual band corresponding to core was quantified by Image J , and calibration curves were built (D). Click here for file

Additional file 2 **Summary on core-specific immune responses in BALB/c and C57BL/6 mice**. Summarized data of immunization experiments performed in BALB/c and C57BL6 mice. The empty vector immunized group and the control group are composed of a mixture of BALB/c (n = 7) and C57BL6 (n = 12) mice. All the other groups had been described in Figures 4 to 6. Click here for file
