# Supplementary figures and images for: Enhancement of the expression of HCV core gene does not enhance core-specific immune response in DNA immunization: advantages of the heterologous DNA prime, protein boost immunization regimen
Source: Genet Vaccines Ther. 2009 Jun 8;7:7. doi: 10.1186/1479-0556-7-7 (PMC2702340; doi:10.1186/1479-0556-7-7)

## Slide 1
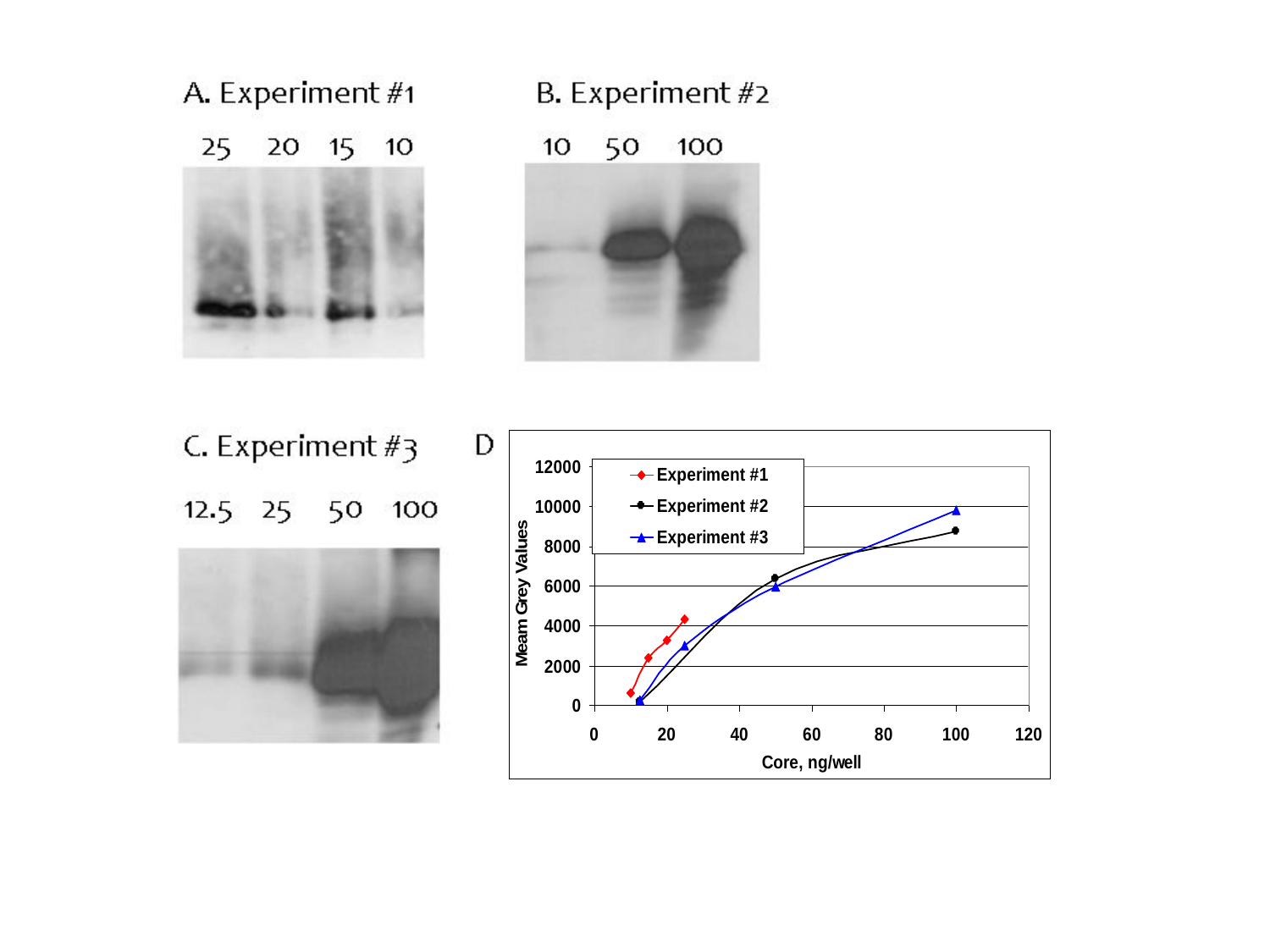

Supplement: Additional file 1 — Establishment of calibration curves for quantification of core expression in vitro. Recombinant core aa 1–173 in serial dilutions in the range of 10 to 25 ng (A), 10 to 100 ng (B), or 12.5 to 100 ng (C) was loaded on 15% SDS-PAAG and resolved by gel electrophoresis together with the study samples. Proteins were transferred to PVDF membrane and subjected to Western blotting with core-specific rabbit antibodies, and secondary anti-rabbit HRP-conjugated antibodies (DAKOPatts). Signals were registered using X-ray films and ECL detection system, images were saved, scanned, and signal of individual band corresponding to core was quantified by Image J , and calibration curves were built (D). [file 1479-0556-7-7-S1.ppt]
