# Supplementary material for: Enhancement of the expression of HCV core gene does not enhance core-specific immune response in DNA immunization: advantages of the heterologous DNA prime, protein boost immunization regimen
Source: Genet Vaccines Ther. 2009 Jun 8;7:7. doi: 10.1186/1479-0556-7-7 (PMC2702340; doi:10.1186/1479-0556-7-7)
Supplement: Additional file 2 — Summary on core-specific immune responses in BALB/c and C57BL/6 mice. Summarized data of immunization experiments performed in BALB/c and C57BL6 mice. The empty vector immunized group and the control group are composed of a mixture of BALB/c (n = 7) and C57BL6 (n = 12) mice. All the other groups had been described in Figures 4 to 6. [file 1479-0556-7-7-S2.ppt]

## Slide 1
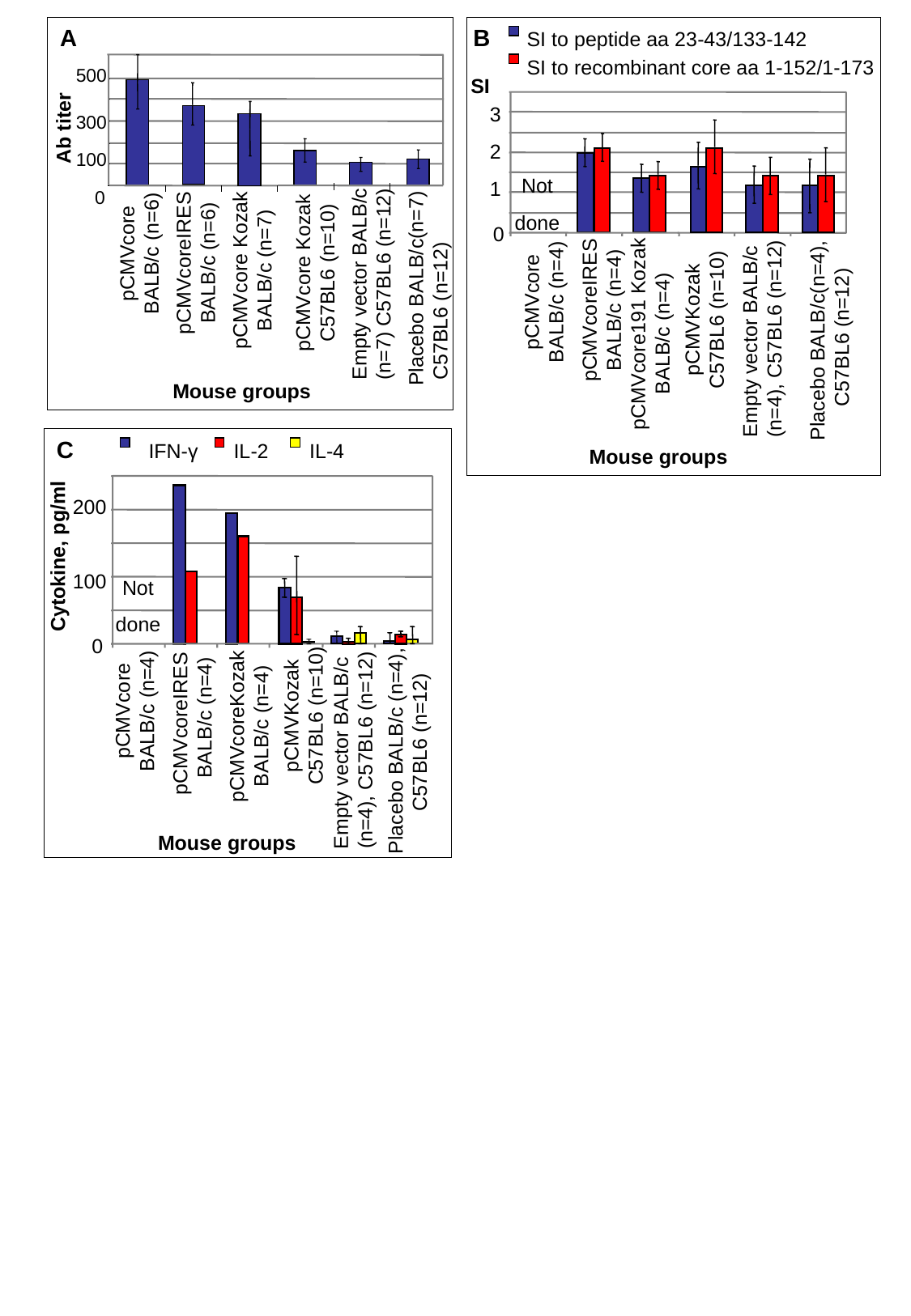

A
B
SI to peptide aa 23-43/133-142
SI to recombinant core aa 1-152/1-173
500
SI
3
Ab titer
300
2
100
Not
done
1
0
0
pCMVcore
BALB/c (n=6)
pCMVcoreIRES
BALB/c (n=6)
pCMVcore Kozak
C57BL6 (n=10)
pCMVcore Kozak
BALB/c (n=7)
Empty vector BALB/c (n=7) C57BL6 (n=12)
Placebo BALB/c(n=7)
 C57BL6 (n=12)
pCMVcore
BALB/c (n=4)
pCMVcoreIRES
BALB/c (n=4)
pCMVKozak
C57BL6 (n=10)
Empty vector BALB/c
(n=4), C57BL6 (n=12)
pCMVcore191 Kozak
BALB/c (n=4)
Placebo BALB/c(n=4),
 C57BL6 (n=12)
Mouse groups
C
Mouse groups
IFN-γ
IL-2
IL-4
200
Cytokine, pg/ml
100
Not
done
0
pCMVKozak
C57BL6 (n=10)
pCMVcore
BALB/c (n=4)
pCMVcoreIRES
BALB/c (n=4)
pCMVcoreKozak
BALB/c (n=4)
Empty vector BALB/c
 (n=4), C57BL6 (n=12)
Placebo BALB/c (n=4),
 C57BL6 (n=12)
Mouse groups
